# The Galerkin Method for Solving Strongly Nonlinear Oscillators

**DOI:** 10.1155/2022/8141227

**Published:** 2022-09-07

**Authors:** Alvaro H. S. Salas

**Affiliations:** Universidad Nacional de Colombia, Fizmako Research Group, Bogotá, Colombia

## Abstract

In this paper, we make use of the Galerkin method for solving nonlinear second-order ODEs that are related to some strongly nonlinear oscillators arising in physics and engineering. We derive the iterative schemes for finding the coefficients that appear in the linear Galerkin hat combination in the ansatz form solution. These coefficients may be found iteratively by solving either a quadratic or a higher degree algebraic equation. Examples are presented to illustrate the obtained results. Some exact solutions are given, and they are compared with both the Runge–Kutta numerical solution and the solution obtained using the Galerkin finite element method.

## 1. Introduction

The nonlinear equation describing an oscillator with a cubic nonlinearity is called the Duffing equation. This equation has a variety of applications in science and engineering, early mechanical failure signal, nonlinear circuit design [1], image processing [2], vibration of buckled beams [3], solitons [4–6], chaos [7], and many areas of physics.

There are many methods for solving nonlinear differential equations. In this paper, we concentrate on the numerical solution to the Duffing equation by means of the finite element method. This method is due to Galerkin, a Russian engineer and scientist. We also derive formulas for solving a wide class of nonlinear oscillators. The Galerkin solutions are compared with the solutions obtained using the Runge–Kutta numerical method.

## 2. Finite Element Method or Galerkin Hat Method

Let us consider a polynomial second-order damped and forced ode:

(1) $$\begin{array}{c} \left\{\begin{array}{c} \ddot{x}+2\varepsilon \dot{x}+P\left(x\right)=f\left(t\right),\\ x\left(0\right)=0,x^{\prime }\left(0\right)={\dot{x}}_{0}, \end{array}\right. \end{array}$$

where *x* ≡ *x*(*t*) and *P* ≡ *P*(*x*) is a polynomial whose coefficients depend on *t*, say

(2) $$\begin{array}{c} P\left(x\right)=\sum_{s=0}^{M}a_{s}\left(t\right)x^{s},x=x\left(t\right). \end{array}$$

Given the i.v.p.

(3) $$\begin{array}{c} \left\{\begin{array}{c} \ddot{y}+2\varepsilon \dot{y}+Q\left(y\right)=f\left(t\right),\\ y\left(0\right)=y_{0}\neq 0,\text{and}y^{\prime }\left(0\right)={\dot{y}}_{0},\\ \text{where}\\ Q\left(y\right)=\sum_{r=0}^{M}b_{r}\left(t\right)y^{r},y=y\left(t\right). \end{array}\right. \end{array}$$

we define

(4) $$\begin{array}{c} y\left(t\right)=y_{0}+x\left(t\right),\\ x\left(0\right)=0,\\ x^{\prime }\left(0\right)=y_{0}. \end{array}$$

Then,

(5) $$\begin{array}{c} Q\left(y\right)={\sum_{r=0}^{M}b_{r}\left(y_{0}+x\left(t\right)\right)}^{r}=\sum_{r=0}^{M}b_{r}\sum_{s=0}^{r}\left(\begin{array}{c} r\\ s \end{array}\right)y_{0}^{r-s}x^{s}\left(t\right)=\sum_{s=0}^{M}\left(\sum_{r=s}^{M}\left(\begin{array}{c} r\\ s \end{array}\right)y_{0}^{r-s}b_{r}\right)x^{s}\left(t\right),\\ =\sum_{s=0}^{M}a_{s}x^{s}≕P\left(x\right),x=x\left(t\right),\\ \text{where}\\ a_{s}=\sum_{r=s}^{M}\left(\begin{array}{c} r\\ s \end{array}\right)y_{0}^{r-s}b_{r}. \end{array}$$

Thus, the problem reduces to (1). Some particular cases to i.v.p. (1) are

(6) $$\begin{array}{c} \left\{\begin{array}{c} \ddot{x}+2\varepsilon \dot{x}+n\left(t\right)+p\left(t\right)x=f\left(t\right),\\ \ddot{x}+2\varepsilon \dot{x}+n\left(t\right)+p\left(t\right)x+q\left(t\right)x^{2}=f\left(t\right),\\ \ddot{x}+2\varepsilon \dot{x}+n\left(t\right)+p\left(t\right)x+q\left(t\right)x^{2}+r\left(t\right)x^{3}=f\left(t\right). \end{array}\right. \end{array}$$

We will use the same idea as for the linear case

(7) $$\begin{array}{c} \left\{\begin{array}{c} \ddot{x}+2\varepsilon \dot{x}+p\left(t\right)x=f\left(t\right),\\ x\left(0\right)=0,x^{\prime }\left(0\right)={\dot{x}}_{0}, \end{array}\right. \end{array}$$

that is, we will assume an approximate analytical solution in the ansatz form

(8) $$\begin{array}{c} x=x\left(t\right)=\sum_{k=1}^{n}c_{k}\varphi_{k}\left(t\right), \end{array}$$

where the functions *φ*_*k*_(*t*) are the so-called linear Galerkin hats. Let 0 ≤ *t* ≤ *T*. Choose some positive integer *n* ≥ 2 and define the step *h*=*T*/*n* and let *ξ*_*j*_=*jh*=*jT*/*n* for *j*=0,1,2,… The functions *φ*_*k*_(*t*) are defined as follows.

(9) $$\begin{array}{c} \varphi_{k}\left(t\right)=\left\{\begin{array}{c} \frac{t-\xi_{k-1}}{h}\;if\;\xi_{k-1}\leq t\leq \xi_{k},\\ -\frac{t-\xi_{k+1}}{h}\;if\;\xi_{k}\leq t\leq \xi_{k+1}, \end{array}\right.\;\text{for}k=1,2,\ldots ,n. \end{array}$$

**Figure 1:**
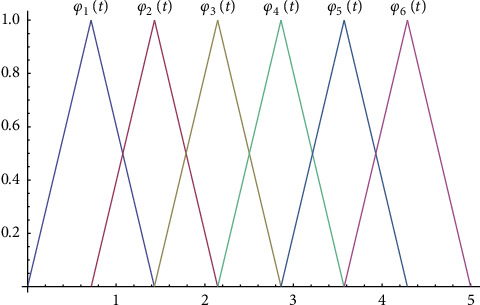
Galerkin hats for *n*=7 and *T*=5.

For an illustration, see Figure 1.

Some properties of these functions can be illustrated as follows.

(10) $$\begin{array}{c} \varphi_{j}\left(t\right)\varphi_{k}\left(t\right)\text{d}t=0, \end{array}$$

for |*j−k*| ≥ 2 and *t* ∈ [0, *T*], and

(11) $$\begin{array}{c} \int_{0}^{T}\varphi_{j}^{p}\left(t\right)=\frac{2T}{\left(p+1\right)n}, \end{array}$$

for *j* ≥ 1 and *p*=1,2,3,….

In general,

(12) $$\begin{array}{c} \int_{0}^{T}\varphi_{j}^{r}\left(t\right)\varphi_{k}^{s}\left(t\right)=\frac{Tr!s!}{n\left(r+s+1\right)!},\;\text{for}\left|j-k\right|=1,\\ r,s=0,1,2,3,\ldots . \end{array}$$

Using the formula

(13) $$\begin{array}{c} \int_{0}^{T}\varphi_{j}\left(t\right)x{\left(t\right)}^{N}\text{d}t=\frac{h}{\left(N+1\right)\left(N+2\right)}\left(\sum_{k=0}^{N-1}\left(k+1\right)\left(c_{j-1}^{N-k}+c_{j+1}^{N-k}\right)c_{j}^{k}+2\left(N+1\right)c_{j}^{N}\right)\text{for any }N\geq 0,\\ c_{0}=c_{n+1}=0, \end{array}$$

and assuming that *a*_*j*_(*t*) ≡ *a*_*j*_= const, we may evaluate easily the following integral:

(14) $$\begin{array}{c} \int_{0}^{T}P\left(x\right)\varphi_{j}\left(t\right)\text{d}t=\sum_{N=0}^{m}a_{N}\int_{0}^{T}\varphi_{j}\left(t\right)x{\left(t\right)}^{N}\text{d}t\text{ for any }j\text{.} \end{array}$$

Moreover,

(15) $$\begin{array}{c} \int_{0}^{T}x\prime \left(t\right)\varphi_{j}\left(t\right)\text{d}t=-\int_{0}^{T}x\prime \left(t\right)\varphi_{j}^{\prime }\left(t\right)\text{d}t=\frac{c_{j-1}-2c_{j}+c_{j+1}}{h}\text{ for any }j,\\ \int_{0}^{T}x\prime \left(t\right)\varphi_{j}\left(t\right)\text{d}t=\frac{1}{2}\left(c_{j+1}-c_{j-1}\right). \end{array}$$

Thus, for example, if *a*_0_, *a*_1_,…are independent of *t*,

(16) $$\begin{array}{c} \left.\int_{0}^{T}\left[x^{\prime \prime }\left(t\right)+\delta x^{\prime }\left(t\right)+a_{0}+a_{1}x\left(t\right)+a_{2}x^{2}\left(t\right)+a_{3}x^{3}\left(t\right)+a_{4}x^{4}\left(t\right)+a_{5}x^{5}\left(t\right)\right]\varphi_{j}\left(t\right)\text{d}t=\frac{n\left(c_{j-1}-2c_{j}+c_{j+1}\right)}{T}+\frac{\delta }{2}\left(c_{j+1}-c_{j-1}\right)+\frac{T}{n}a_{0}+a_{1}A_{1}+a_{2}A_{2}+a_{3}A_{3}+a_{4}A_{4}+a_{5}A_{5}\right), \end{array}$$

where

(17) $$\begin{array}{c} A_{1}=\frac{T}{6n}\left(c_{j-1}+4c_{j}+c_{j+1}\right),\\ A_{2}=\frac{T}{12n}\left(c_{j-1}^{2}+2c_{j}c_{j-1}+6c_{j}^{2}+c_{j+1}^{2}+2c_{j}c_{j+1}\right),\\ A_{3}=\frac{T}{20n}\left(c_{j-1}^{3}+2c_{j}c_{j-1}^{2}+3c_{j}^{2}c_{j-1}+8c_{j}^{3}+c_{j+1}^{3}+2c_{j}c_{j+1}^{2}+3c_{j}^{2}c_{j+1}\right),\\ A_{4}=\frac{T}{30n}\left(c_{j-1}^{4}+2c_{j}c_{j-1}^{3}+3c_{j}^{2}c_{j-1}^{2}+4c_{j}^{3}c_{j-1}+10c_{j}^{4}+c_{j+1}^{4}+2c_{j}c_{j+1}^{3}+3c_{j}^{2}c_{j+1}^{2}+4c_{j}^{3}c_{j+1}\right),\\ A_{5}=\frac{T}{42n}\left(c_{j-1}^{5}+2c_{j}c_{j-1}^{4}+3c_{j}^{2}c_{j-1}^{3}+4c_{j}^{3}c_{j-1}^{2}+5c_{j}^{4}c_{j-1}+12c_{j}^{5}+c_{j+1}^{5}+2c_{j}c_{j+1}^{4}+3c_{j}^{2}c_{j+1}^{3}+4c_{j}^{3}c_{j+1}^{2}+5c_{j}^{4}c_{j+1}\right). \end{array}$$

In general, if *a*_*r*_ does not depend on *t*,

(18) $$\begin{array}{c} \int_{0}^{T}\left(x^{\prime \prime }\left(t\right)+\delta x^{\prime }\left(t\right)+\sum_{s=0}^{M}a_{s}x^{s}\left(t\right)\right)\varphi_{j}\left(t\right)\text{d}t=\frac{n\left(c_{j-1}-2c_{j}+c_{j+1}\right)}{T}+\frac{1}{2}\delta \left(c_{j+1}-c_{j-1}\right)+\frac{Ta_{0}}{n}+\sum_{s=0}^{M}A_{s}a_{s}, \end{array}$$

where

(19) $$\begin{array}{c} A_{s}=\frac{T}{n\left(s+1\right)\left(s+2\right)}\sum_{k=0}^{s-1}\left(\left(k+1\right)\left(c_{j-1}^{s-k}+c_{j+1}^{s-k}\right)c_{j}^{k}+2\left(s+1\right)c_{j}^{s}\right). \end{array}$$

Let us consider the forced and damped oscillator (1). Following are useful expressions for different forces:

(20) $$\begin{array}{c} \int_{0}^{T}f\left(t\right)\varphi_{j}\left(t\right)\text{d}t=\frac{4n}{T\Omega^{2}}\sin^{2}\left(\frac{T\Omega }{2n}\right)\left(\frac{F_{0}T}{n}+F_{1}\cos\left(\frac{jT\Omega }{n}\right)+F_{2}\sin\left(\frac{jT\Omega }{n}\right)\right),\\ f\left(t\right)=f_{0}+f_{1}\cos\left(\Omega t\right)+f_{2}\sin\left(\Omega t\right),\\ \Omega \neq 0,\\ \int_{0}^{T}f\left(t\right)\varphi_{j}\left(t\right)\text{d}t=\frac{ne^{-\left(j+1\right)\lambda T/n}}{T{\left(\lambda^{2}+\Omega^{2}\right)}^{2}}\left(\begin{array}{c} -2\lambda \Omega \sin\left(\frac{\left(j+1\right)T\Omega }{n}\right)+\left(\lambda -\Omega \right)\left(\lambda +\Omega \right)e^{\lambda T/n}\cos\left(\frac{\left(j-1\right)T\Omega }{n}\right)+\\ \left(\lambda -\Omega \right)\left(\lambda +\Omega \right)\cos\left(\frac{\left(j+1\right)T\Omega }{n}\right)-\\ 2e^{\lambda T/n}\left(\lambda \Omega \left(e^{\lambda T/n}\sin\left(\frac{\left(j-1\right)T\Omega }{n}\right)-2\sin\left(\frac{jT\Omega }{n}\right)\right)+\left(\lambda -\Omega \right)\left(\lambda +\Omega \right)\cos\left(\frac{jT\Omega }{n}\right)\right) \end{array}\right),\\ f\left(t\right)=e^{-\lambda t}\cos\left(\Omega t\right),\\ {\left|\Omega \right|}^{2}+{\left|\lambda \right|}^{2}\neq 0,\\ \int_{0}^{T}f\left(t\right)\varphi_{j}\left(t\right)\text{d}t=\frac{ne^{-\left(j+1\right)\lambda T/n}}{T{\left(\lambda^{2}+\Omega^{2}\right)}^{2}}\left(\begin{array}{c} \left(\lambda -\Omega \right)\left(\lambda +\Omega \right)e^{\lambda T/n}\left(e^{\lambda T/n}\sin\left(\frac{\left(j-1\right)T\Omega }{n}\right)-2\sin\left(\frac{jT\Omega }{n}\right)\right)+\\ \left(\lambda -\Omega \right)\left(\lambda +\Omega \right)\sin\left(\frac{\left(j+1\right)T\Omega }{n}\right)+2\lambda \Omega e^{2\lambda T/n}\cos\left(\frac{\left(j-1\right)T\Omega }{n}\right)-\\ 4\lambda \Omega e^{\lambda T/n}\cos\left(\frac{jT\Omega }{n}\right)+2\lambda \Omega \cos\left(\frac{\left(j+1\right)T\Omega }{n}\right) \end{array}\right),\\ f\left(t\right)=e^{-\lambda t}\sin\left(\Omega t\right),\\ {\left|\Omega \right|}^{2}+{\left|\lambda \right|}^{2}\neq 0,\\ \int_{0}^{T}f\left(t\right)\varphi_{j}\left(t\right)\text{d}t=\frac{\lambda^{-\rho -2}}{T}\left(\begin{array}{c} \lambda T\left(\left(1-j\right)\Gamma \left(\rho +1,\frac{\left(j-1\right)T\lambda }{n}\right)+2j\Gamma \left(\rho +1,\frac{jT\lambda }{n}\right)-\left(j+1\right)\Gamma \left(\rho +1,\frac{\left(j+1\right)T\lambda }{n}\right)\right)+\\ n\left(\Gamma \left(\rho +2,\frac{\left(j-1\right)T\lambda }{n}\right)-2\Gamma \left(\rho +2,\frac{jT\lambda }{n}\right)+\Gamma \left(\rho +2,\frac{\left(j+1\right)T\lambda }{n}\right)\right) \end{array}\right),\\ f\left(t\right)=e^{-\lambda t}t^{\rho },\\ \int_{0}^{T}f\left(t\right)\varphi_{j}\left(t\right)dt\approx \frac{n}{9Tw}\left(\left(A-1\right)\left(\cos\left(\frac{3\left(j-1\right)T\sqrt{w}}{n}\right)-2\cos\left(\frac{3jT\sqrt{w}}{n}\right)+\cos\left(\frac{3\left(j+1\right)T\sqrt{w}}{n}\right)\right)-18A\left(\cos\left(\frac{T\sqrt{w}}{n}\right)-1\right)\cos\left(\frac{jT\sqrt{w}}{n}\right)\right),\\ f\left(t\right)=cn\left(\Omega ,m\right)\approx A\cos\left(\sqrt{w}t\right)+\left(1-A\right)\cos\left(3\sqrt{w}t\right).w=\frac{\left(27m^{2}-92m+64\right)\Omega^{2}}{3m^{2}-60m+64}.A=\frac{2\left(15m^{2}-48m+32\right)}{27m^{2}-92m+64},\left|m\right|\leq 0.5,\\ \int_{0}^{T}f\left(t\right)\varphi_{j}\left(t\right)dt\approx \frac{n}{9Tw}\left(\left(A-1\right)\left(\cos\left(\frac{3\left(j-1\right)T\sqrt{w}}{n}\right)-2\cos\left(\frac{3jT\sqrt{w}}{n}\right)+\cos\left(\frac{3\left(j+1\right)T\sqrt{w}}{n}\right)\right)-18A\left(\cos\left(\frac{T\sqrt{w}}{n}\right)-1\right)\cos\left(\frac{jT\sqrt{w}}{n}\right)\right),\\ f\left(t\right)=cn\left(\Omega t,m\right)\approx A\cos\left(\sqrt{w}t\right)+\left(1-A\right)\cos\left(3\sqrt{w}t\right).w\\ =\frac{\left(27m^{2}-92m+64\right)\Omega^{2}}{3m^{2}-60m+64}.A=\frac{2\left(15m^{2}-48m+32\right)}{27m^{2}-92m+64},\;\left|m\right|\leq 0.5,\\ \int_{0}^{T}f\left(t\right)\varphi_{j}\left(t\right)\text{d}t\\ \approx \frac{{\left(3-4A\right)}^{2}n\left(18A\left(\cos\left(T\Omega /3n-4An\right)-1\right)\sin\left(jT\Omega /3n-4An\right)-\left(A-1\right)\left(\sin\left(3\left(j-1\right)T\Omega /\left(4A-3\right)n\right)+\sin\left(3\left(j+1\right)T\Omega /\left(4A-3\right)n\right)+2\sin\left(3jT\Omega /3n-4An\right)\right)\right)}{9T\Omega^{2}},\\ f\left(t\right)=sn\left(\Omega t,m\right)\approx A\sin\left(\frac{t\Omega }{4A-3}\right)-\left(1-A\right)\sin\left(\frac{3t\Omega }{4A-3}\right),\\ A=-\frac{-25m^{2}+144m-128}{29m^{2}-152m+128},\;\left|m\right|\leq 0.25. \end{array}$$

Other useful formulas when the *a*_*j*_ depend on time are

(21) $$\begin{array}{c} \int_{0}^{T}tx\left(t\right)\varphi_{j}\left(t\right)\text{d}t=\frac{1}{12}h\left(c_{j-1}\left(2a+h\left(2j-1\right)\right)+8c_{j}\left(a+hj\right)+c_{j+1}\left(2a+2hj+h\right)\right),\\ \int_{0}^{T}t^{2}x\left(t\right)\varphi_{j}\left(t\right)\text{d}t=\frac{1}{60}h^{3}\left(\left(40j^{2}+4\right)c_{j}+\left(10\left(j-1\right)j+3\right)c_{j-1}+\left(10j\left(j+1\right)+3\right)c_{j+1}\right),\\ \int_{0}^{T}t^{3}x\left(t\right)\varphi_{j}\left(t\right)\text{d}t=\frac{1}{60}h^{4}\left(4j\left(10j^{2}+3\right)c_{j}+\left(2j-1\right)\left(5\left(j-1\right)j+2\right)c_{j-1}+\left(2j+1\right)\left(5j\left(j+1\right)+2\right)c_{j+1}\right),\\ \int_{0}^{T}tx{\left(t\right)}^{2}\varphi_{j}\left(t\right)\text{d}t=\frac{1}{60}h^{2}\left(\left(5j-3\right)c_{j-1}^{2}+2\left(5j-2\right)c_{j}c_{j-1}+30jc_{j}^{2}+\left(5j+3\right)c_{j+1}^{2}+2\left(5j+2\right)c_{j}c_{j+1}\right),\\ \int_{0}^{T}t^{2}x{\left(t\right)}^{2}\varphi_{j}\left(t\right)\text{d}t=\frac{1}{60}h^{3}\left(\begin{array}{c} \left(30j^{2}+2\right)c_{j}^{2}+\left(j\left(5j-6\right)+2\right)c_{j-1}^{2}+\\ 2\left(j\left(5j-4\right)+1\right)c_{j}c_{j-1}+\left(j\left(5j+6\right)+2\right)c_{j+1}^{2}+2\left(j\left(5j+4\right)+1\right)c_{j}c_{j+1} \end{array}\right),\\ \int_{0}^{T}t^{3}x{\left(t\right)}^{2}\varphi_{j}\left(t\right)dt=\frac{1}{420}h^{4}\left(\begin{array}{c} 42\left(5j^{3}+j\right)c_{j}^{2}+7j\left(j\left(5j-9\right)+6\left.-\right)10\right)c_{j-1}^{2}+2\left(7j\left(j\left(5j-6\right)+3\right)-4\right)c_{j}c_{j-1}+\\ \left(7j\left(j\left(5j+9\right)+6\right)+10\right)c_{j+1}^{2}+2\left(7j\left(j\left(5j+6\right)+3\right)+4\right)c_{j}c_{j+1} \end{array}\right),\\ \int_{0}^{T}tx{\left(t\right)}^{3}\varphi_{j}\left(t\right)\text{d}t=\frac{1}{60}h^{2}\left(\begin{array}{c} \left(3j-2\right)c_{j-1}^{3}+3\left(2j-1\right)c_{j}c_{j-1}^{2}+3\left(3j-1\right)c_{j}^{2}c_{j-1}+\\ 24jc_{j}^{3}+\left(3j+2\right)c_{j+1}^{3}+3\left(2j+1\right)c_{j}c_{j+1}^{2}+3\left(3j+1\right){}_{\left.j\right)}^{2}c_{j+1} \end{array}\right),\\ \int_{0}^{T}t^{2}x{\left(t\right)}^{3}\varphi_{j}\left(t\right)\text{d}t=\frac{1}{420}h^{3}\left(\begin{array}{c} 8\left(21j^{2}+1\right)c_{j}^{3}+\left(7j\left(3j-4\right)+10\right)c_{j-1}^{3}+6\left(7\left(j-1\right)j+2\right)c_{j}c_{j-1}^{2}+\\ 3\left(7j\left(3j-2\right)+3\right)c_{j}^{2}c_{j-1}+\left(7j\left(3j+4\right)+10\right)c_{j+1}^{3}+\\ 6\left(7j\left(j+1\right)+2\right)c_{j}c_{j+1}^{2}+3\left(7j\left(3j+2\right)+3\right)c_{j}^{2}c_{j+1} \end{array}\right),\\ \int_{0}^{T}t^{3}x{\left(t\right)}^{3}\varphi_{j}\left(t\right)\text{d}t=\frac{1}{280}h^{4}\left(\begin{array}{c} 16\left(7j^{3}+j\right)c_{j}^{3}+{\left(2j\left(7\left(j-2\right)j+10\right)-5\right)}_{j-1}^{3}+\left(2j-1\right)\left(14\left(j-1\right)j+5\right)c_{j}c_{j-1}^{2}+\\ 3\left(2j\left(7\left(j-1\right)j+3\right)-1\right)c_{j}^{2}c_{j-1}+\left(2j\left(7j\left(j+2\right)+10\right)+5\right)c_{j+1}^{3}+\\ \left(2j+1\right)\left(14j\left(j+1\right)+5\right)c_{j}c_{j+1}^{2}+3\left(2j\left(7j\left(j+1\right)+3\right)+1\right)c_{j}^{2}c_{j+1} \end{array}\right). \end{array}$$

Other formulas for calculating are given in the Appendix.

## 3. Applications

### 3.1. Linear Oscillator

This is the ode

(22) $$\begin{array}{c} \ddot{x}+\alpha +\beta x=0,\\ \beta \neq 0,\\ x\left(0\right)=0,\\ x^{\prime }\left(0\right)={\dot{x}}_{0\;},\\ 0\leq t\leq T. \end{array}$$

The exact solution is given by

(23) $$\begin{array}{c} x\left(t\right)=\frac{1}{\beta }\left(\alpha \left(\cos\left(\sqrt{\beta }t\right)-1\right)+\sqrt{\beta }{\dot{x}}_{0}\sin\left(\sqrt{\beta }t\right)\right). \end{array}$$

Assume the ansatz

(24) $$\begin{array}{c} x=x\left(t\right)=\sum_{k=1}^{n}c_{k}\varphi_{k}\left(t\right). \end{array}$$

Define $c_{-1}=c_{0}=0,c_{1}=\left(T\;/n\right){\dot{x}}_{0}$ and

(25) $$\begin{array}{c} R_{j}=\int_{0}^{T}\left(\ddot{x}+\alpha +\beta x\right)\varphi_{j}\left(t\right)\text{d}t,\;j=0,1,2,\ldots ,n-1. \end{array}$$

Then,

(26) $$\begin{array}{c} R_{j}=\frac{6\alpha T^{2}}{6nT}+\frac{6n^{2}+\beta T^{2}}{6nT}c_{j-1}+\frac{4c\left(\beta T^{2}-3n^{2}\right)}{6nT}c_{j}+\left(\frac{\beta T}{6n}+\frac{n}{T}\right)c_{j+1}=0\text{ for any }j. \end{array}$$

This is a linear recurrence that may be solved in closed form:

(27) $$\begin{array}{c} c_{j}=\frac{1}{6\beta }\left(\begin{array}{c} -6\alpha +3\alpha {\left(\frac{T\left(\sqrt{3}\sqrt{\beta \left(\beta T^{2}-12n^{2}\right)}-2\beta T\right)+6n^{2}}{6n^{2}+\beta T^{2}}\right)}^{j}+\\ 3\alpha {\left(\frac{6n^{2}-T\left(\sqrt{3}\sqrt{\beta \left(\beta T^{2}-12n^{2}\right)}+2\beta T\right)}{6n^{2}+\beta T^{2}}\right)}^{j}+\\ \frac{1}{n\sqrt{T^{2}/3-4n^{2}/\beta }}\left(\begin{array}{c} \left(\begin{array}{c} {\left(T\left(\sqrt{3}\sqrt{\beta \left(\beta T^{2}-12n^{2}\right)}-2\beta T\right)+6n^{2}\right)}^{j}-\\ {\left(6n^{2}-T\left(\sqrt{3}\sqrt{\beta \left(\beta T^{2}-12n^{2}\right)}+2\beta T\right)\right)}^{j}\\ {\left(6n^{2}+\beta T^{2}\right)}^{-j}\left({\dot{x}}_{0}\left(6n^{2}+\beta T^{2}\right)+3\alpha nT\right) \end{array}\right) \end{array}\right),\\ j=1,2,3,\ldots ,n \end{array}\right). \end{array}$$

**Example 1 .**

Let *α*=0, *β*=1, ${\dot{x}}_{0}=1$, and *T*=2*π*. The exact solution to (14) is *x*(*t*)=sin(*t*). From (19), we obtain

(28) $$\begin{array}{c} c_{j}=\frac{\left(\left(17440418\pi^{2}/n^{2}\right)+26160627\right)}{2953\sqrt{78481881-\left(26160627\pi^{2}/n^{2}\right)}}\sin\left(j\tan^{-1}\left(\frac{5906\pi \sqrt{78481881-\left(26160627\pi^{2}/n^{2}\right)}}{n\left(26160627-\left(34880836\pi^{2}/n^{2}\right)\right)}\right)\right). \end{array}$$

**Figure 2:**
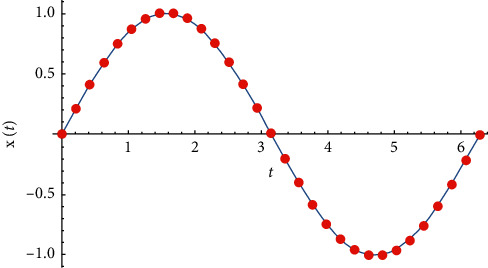
Comparison between the Galerkin solution and the Runge–Kutta numerical solution.

In Figure 2, we compare the exact solution with the approximate solution (pairs (2*π*/*nj*, *c*_*j*_),  *j*=1,2,3,…, *n*) for *n*=30.

### 3.2. Undamped and Unforced Helmholtz Oscillator

This is the ode

(29) $$\begin{array}{c} \ddot{y}+\alpha +\beta y+\gamma y^{2}=0,\\ y\left(0\right)=y_{0},\\ y^{\prime }\left(0\right)={\dot{y}}_{0\;},\\ 0\leq t\leq T. \end{array}$$

The exact solution to (22) may be expressed in any of the following equivalent forms:

(30) $$\begin{array}{c} y\left(t\right)=-\frac{\beta }{2\gamma }-\frac{6℘\left(t+t_{0};\left(1/12\right)\left(\beta^{2}-4\alpha \gamma \right),\left(1/216\right)\left(\left(\beta +2\gamma y_{0}\right)\left(\beta^{2}-6\alpha \gamma -2\gamma y_{0}\left(\beta +\gamma y_{0}\right)\right)-6\gamma^{2}{\dot{y}}_{0}^{2}\right)\right)}{\gamma },\\ \text{where}\\ t_{0}=\pm {℘}^{-1}\left(\left(1/12\right)\left(-\beta -2\gamma y_{0}\right);\left(1/12\right)\left(\beta^{2}-4\alpha \gamma \right),\left(1/216\right)\left(\left(\beta +2\gamma y_{0}\right)\left(-6\alpha \gamma +\beta^{2}-2\gamma y_{0}\left(\beta +\gamma y_{0}\right)\right)-6\gamma^{2}{\dot{y}}_{0}^{2}\right)\right),\\ y\left(t\right)=A-\frac{6\left(\alpha +A\left(A\gamma +\beta \right)\right)}{12℘\left(t+t_{0};1/12\left(\beta^{2}-4\alpha \gamma \right),1/216\left(\beta +2A\gamma \right)\left(\beta^{2}-2A\gamma \beta -2\gamma \left(\gamma A^{2}+3\alpha \right)\right)\right)+2A\gamma +\beta },\\ \text{where}\\ -2\gamma A^{3}+3\beta A^{2}+6\alpha A+6\alpha y_{0}-3\beta y_{0}^{2}-2\gamma y_{0}^{3}-3{\dot{y}}_{0}^{2}=0,\\ t_{0}={℘}^{-1}\left(\frac{6\alpha +4A^{2}\gamma +5A\beta +2A\gamma y_{0}+\beta y_{0}}{12\left(A-y_{0}\right)};\frac{1}{12}\left(\beta^{2}-4\alpha \gamma \right),\frac{1}{216}\left(2A\gamma +\beta \right)\left(-2\gamma \left(3\alpha +A^{2}\gamma \right)-2A\beta \gamma +\beta^{2}\right)\right),\\ y\left(t\right)=A+{\left(\frac{\sqrt{y_{0}-A}cn\left(\sqrt{\omega }t,m\right)+b_{1}sn\left(\sqrt{\omega }t,m\right)dn\left(\sqrt{\omega }t,m\right)}{1+b_{2}sn^{2}\left(\sqrt{\omega }t,m\right)}\right)}^{2}, \end{array}$$

where

(31) $$\begin{array}{c} b_{1}=\frac{{\dot{y}}_{0}}{2\sqrt{\omega }\sqrt{y_{0}-A}},\\ b_{2}=\frac{{\dot{y}}_{0}^{2}-2\left(A-y_{0}\right)\left(\alpha +2A\omega +y_{0}\left(\beta +\gamma y_{0}-2\omega \right)\right)}{8\omega {\left(A-y_{0}\right)}^{2}},\\ \omega =-\frac{-2A^{2}\beta -4A^{2}\gamma y_{0}-6\alpha A-2A\beta y_{0}+2A\gamma y_{0}^{2}+6\alpha y_{0}+4\beta y_{0}^{2}+2\gamma y_{0}^{3}+3{\dot{y}}_{0}^{2}}{4\left(2m-1\right){\left(A-y_{0}\right)}^{2}},\\ m=\frac{1}{2}\left(1-\frac{\sqrt{3}{\left(2A\gamma +\beta \right)}^{2}}{\sqrt{{\left(2A\gamma +\beta \right)}^{2}\left(\left(\beta -2A\gamma \right)\left(2A\gamma +3\beta \right)-16\alpha \gamma \right)}}\right),\\ 2\gamma A^{3}+3\beta A^{2}+6\alpha A-6\alpha y_{0}-3\beta y_{0}^{2}-2\gamma y_{0}^{3}-3{\dot{y}}_{0}^{2}=0. \end{array}$$

The period equals $T=\left(2K\left(m\right)/\sqrt{w}\right)$

In order to obtain approximate solution by means of the Galerkin method, let

(32) $$\begin{array}{c} y\left(t\right)=y_{0}+x\left(t\right),\\ x\left(0\right)=0,\\ x^{\prime }\left(0\right)={\dot{y}}_{0\;}. \end{array}$$

Then,

(33) $$\begin{array}{c} \ddot{x}+\alpha +\beta y_{0}+\gamma y_{0}^{2}+\left(\beta +2\gamma y_{0}\right)x\left(t\right)+\gamma x{\left(t\right)}^{2}=0,\\ x\left(0\right)=0,\\ x^{\prime }\left(0\right)={\dot{y}}_{0\;}. \end{array}$$

Assume the ansatz

(34) $$\begin{array}{c} x=x\left(t\right)=\sum_{k=1}^{n}c_{k}\varphi_{k}\left(t\right). \end{array}$$

Define $c_{-1}=c_{0}=0,c_{1}=T\;/n{\dot{x}}_{0}$ and

(35) $$\begin{array}{c} R_{j}=\int_{0}^{T}\left(\ddot{x}+\alpha +\beta y_{0}+\gamma y_{0}^{2}+\left(\beta +2\gamma y_{0}\right)x\left(t\right)+\gamma x{\left(t\right)}^{2}\right)\varphi_{j}\left(t\right)\text{d}t. \end{array}$$

Then,

(36) $$\begin{array}{c} R_{j}=\frac{8c_{j}\left(-3n^{2}+\beta T^{2}+2\gamma T^{2}y_{0}\right)+2c_{j-1}\left(\gamma T^{2}\left(c_{j}+2y_{0}\right)+6n^{2}+\beta T^{2}\right)+\gamma T^{2}c_{j-1}^{2}+6\gamma T^{2}c_{j}^{2}+12T^{2}\left(\alpha +y_{0}\left(\beta +\gamma y_{0}\right)\right)}{12nT}\\ +\frac{\left(\gamma T^{2}\left(c_{j}+2y_{0}\right)+6n^{2}+\beta T^{2}\right)}{6nT}c_{j+1}+\frac{\gamma T}{12n}c_{j+1}^{2}=0. \end{array}$$

Observe that the equation *R*_*j*_=0 is a quadratic equation in *z*=*c*_*j*+1_. It is clear that the system *R*_0_=*R*_1_=…=*R*_*n−*1_=0 may be solved recursively. We first find *c*_2_ letting *j*=1 in (29):

(37) $$\begin{array}{c} c_{2}=\frac{-6n^{2}-T^{2}\left(\beta +2\gamma y_{0}\right)\pm \sqrt{{\left(6n^{2}+\beta T^{2}\right)}^{2}+\gamma T^{2}\left(-2nT{\dot{y}}_{0}\left(6n^{2}+\beta T^{2}+2\gamma T^{2}y_{0}\right)-8n^{2}y_{0}\left(-3n^{2}+\beta T^{2}+\gamma T^{2}y_{0}\right)-\gamma T^{4}{\dot{y}}_{0}^{2}\right)/n^{2}-12\alpha \gamma T^{4}}}{\gamma T^{2}}. \end{array}$$

We choose the value of *c*_2_ that is closest to $c_{1}=T/n{\dot{x}}_{0\;}$. Next, we set *j*=2 in (37) and then we will find *z*=*c*_3_. We choose the closest to *c*_2_ solution to the quadratic equation in (29). We continue this procedure and then we will find all values of *c*_*j*_(*j*=1,2,3,…*n*). Since *x*(*ξ*_*j*_)=*x*(*T*/*nj*)=*c*_*j*_, all pairs (*ξ*_*j*_, *c*_*j*_) will lie on the graph of the solution for sufficiently large *n*. Plotting these points, we obtain the graph of the solution.

On the other hand, if *n* is large enough, the values of *c*_*j*_ and *c*_*j*+1_ will be close to each other. We may use the following approximate expression for *c*_*j*+1_ in terms of *c*_*j−*1_ and *c*_*j*_:

(38) $$\begin{array}{c} c_{j+1}\approx -\frac{8c_{j}\left(-3n^{2}+\beta T^{2}+2\gamma T^{2}y_{0}\right)+2c_{j-1}\left(\gamma T^{2}\left(c_{j}+2y_{0}\right)+6n^{2}+\beta T^{2}\right)+\gamma T^{2}c_{j-1}^{2}+5\gamma T^{2}c_{j}^{2}+12T^{2}\left(\alpha +y_{0}\left(\beta +\gamma y_{0}\right)\right)}{2\left(2\gamma T^{2}\left(c_{j}+y_{0}\right)+6n^{2}+\beta T^{2}\right)}. \end{array}$$

**Example 2 .**

Let

(39) $$\begin{array}{c} \ddot{y}+2y\left(t\right)-y^{2}\left(t\right)=0\wedge y\left(0\right)=0\wedge y^{\prime }\left(0\right)=1. \end{array}$$

The exact solution is obtained from (25) and (26):

(40) $$\begin{array}{c} y\left(t\right)=-0.641784+\frac{{\left(\left(0.801114cn\left(0.574208\right.t|-0.744849\right)+1.08694dn\left(0.574208t|-0.744849\right)sn\left(0.574208t|-0.744849\right.\right)}^{2}}{{\left(1+0.420436sn{\left(0.574208t|-0.744849\right)}^{2}\right)}^{2}}. \end{array}$$

**Figure 3:**
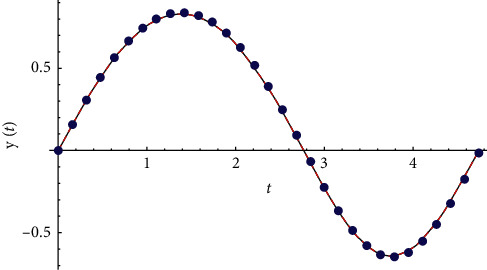
Comparison between the exact solution and the finite element method for *n*=30.

The solution is periodic and its period equals *T*=4.737423705838371 (see Figure 3).

### 3.3. Duffing–Helmholtz Oscillator

Let

(41) $$\begin{array}{c} \ddot{q}+\alpha +\beta q+\gamma q^{2}+\delta q^{3}=0,\\ q\left(0\right)=q_{0},\\ q^{\prime }\left(0\right)={\dot{q}}_{0}. \end{array}$$

The exact solution to i.v.p. (34) is given by

(42) $$\begin{array}{c} q\left(t\right)=A+\frac{B}{1+C℘\left(t+t_{0};g_{2},g_{3}\right)}, \end{array}$$

where

(43) $$\begin{array}{c} B=-\frac{6\left(A^{3}\delta +A^{2}\gamma +A\beta +\alpha \right)}{3A^{2}\delta +2A\gamma +\beta },\\ C=\frac{12}{3A^{2}\delta +2A\gamma +\beta },\\ t_{0}=\pm {℘}^{-1}\left(\frac{q_{0}-A-B}{C\left(A-q_{0}\right)};g_{2},g_{3}\right),\\ g_{2}=\frac{1}{12}\left(-3A^{4}\delta^{2}-4A^{3}\gamma \delta -6A^{2}\beta \delta -12A\alpha \delta -4\alpha \gamma +\beta^{2}\right),\\ g_{3}=\frac{1}{216}\left(-3A^{4}\delta \left(\gamma^{2}-3\beta \delta \right)-4A^{3}\gamma \left(\gamma^{2}-3\beta \delta \right)+6A^{2}\beta \left(3\beta \delta -\gamma^{2}\right)-12A\alpha \left(\gamma^{2}-3\beta \delta \right)+27\alpha^{2}\delta -6\alpha \beta \gamma +\beta^{3}\right),\\ 3\delta A^{4}+4\gamma A^{3}+6\beta A^{2}+12A\alpha -\left(12q_{0}\alpha +6q_{0}^{2}\beta +4q_{0}^{3}\gamma +3q_{0}^{4}\delta +6{\dot{q}}_{0}^{2}\right)=0. \end{array}$$

This solution is valid even if *α*=*γ*=0. The solution is periodic and its period equals

(44) $$\begin{array}{c} T=2\int_{a}^{\infty }\frac{1}{\sqrt{4x^{3}-g_{2}x-g_{3}}}, \end{array}$$

where *a* is the greatest real root to the cubic 4*x*^3^*−g*_2_*x−g*_3_=0. Assume that 4*x*^3^*−g*_2_*x−g*_3_=4(*x−a*)(*x−b*)(*x−c*). Then, the period may be evaluated using the formulas

(45) ∫audxx−ax−bx−c=2c−bFsin−1b−cu−ab−au−c,b−ab−c,∫a∞dxx−ax−bx−c=limu⟶∞2c−bFsin−1b−cu−ab−au−c,b−ab−c=2c−bKb−ab−c,∫audxx−ax−bx−b¯=Fa−α2+β24cos−1a−u+a−α2+β2−a+u+a−α2+β2,α−a+a−α2+β22a−α2+β2,∫a∞dxx−ax−bx−b¯=limu⟶∞∫audxx−ax−bx−b¯=2a−α2+β24K−a+α+a−α2+β22a−α2+β2,α=Reb,β=Imb.

Another expression for the exact solution is given by

(46) $$\begin{array}{c} q\left(t\right)=A+\frac{B}{1+v_{0}cn\left(\sqrt{a+bv_{0}^{2}}t,bv_{0}^{2}/2\left(a+bv_{0}^{2}\right)\right)}, \end{array}$$

where

(47) $$\begin{array}{c} v_{0}=\frac{q_{0}-A-B}{A-q_{0}},\\ 3\delta A^{4}+4\gamma A^{3}+6\beta A^{2}+12\alpha A-\left(12q_{0}\alpha +6q_{0}^{2}\beta +4q_{0}^{3}\gamma +3q_{0}^{4}\delta \right)=0,\\ a=\frac{\begin{array}{c} A^{4}\left(-\left(\gamma^{2}-3\beta \delta \right)\right)-2A^{3}\left(\beta \gamma -9\alpha \delta \right)-3A^{2}\left(12q_{0}\alpha \delta +6q_{0}^{2}\beta \delta +4q_{0}^{3}\gamma \delta +3q_{0}^{4}\delta^{2}-2\alpha \gamma +\beta^{2}\right)-\\ 2A\left(12q_{0}\alpha \gamma +6q_{0}^{2}\beta \gamma +4q_{0}^{3}\gamma^{2}+3q_{0}^{4}\gamma \delta +3\alpha \beta \right)-12q_{0}\alpha \beta -6q_{0}^{2}\beta^{2}-4q_{0}^{3}\beta \gamma -3q_{0}^{4}\beta \delta -9\alpha^{2} \end{array}}{\left(A-q_{0}\right)\left(3A^{3}\delta +A^{2}\left(3q_{0}\delta +4\gamma \right)+A\left(4q_{0}\gamma +3q_{0}^{2}\delta +6\beta \right)+6q_{0}\beta +4q_{0}^{2}\gamma +3q_{0}^{3}\delta +12\alpha \right)},\\ b=-\frac{3{\left(A^{3}\delta +A^{2}\gamma +A\beta +\alpha \right)}^{2}}{\left(A-q_{0}\right)\left(3A^{3}\delta +A^{2}\left(3q_{0}\delta +4\gamma \right)+A\left(4q_{0}\gamma +3q_{0}^{2}\delta +6\beta \right)+6q_{0}\beta +4q_{0}^{2}\gamma +3q_{0}^{3}\delta +12\alpha \right)},\\ B=-\frac{\left(A-q_{0}\right)\left(3A^{3}\delta +A^{2}\left(3q_{0}\delta +4\gamma \right)+A\left(4q_{0}\gamma +3q_{0}^{2}\delta +6\beta \right)+6q_{0}\beta +4q_{0}^{2}\gamma +3q_{0}^{3}\delta +12\alpha \right)}{3\left(A^{3}\delta +A^{2}\gamma +A\beta +\alpha \right)}. \end{array}$$

Let *q*_0_=0. Assume the ansatz

(48) $$\begin{array}{c} q=q\left(t\right)=\sum_{k=1}^{n}c_{k}\varphi_{k}\left(t\right). \end{array}$$

Define *c*_0_=*c*_*n*+1_=0. We have

(49) $$\begin{array}{c} \int_{0}^{T}\left(\left(\ddot{q}t\right)+\alpha +\beta q\left(t\right)+\gamma q^{2}\left(t\right)+\delta q^{3}\left(t\right)\right)\varphi_{j}\left(t\right)\text{d}t=\frac{c_{j-1}-2c_{j}+c_{j+1}}{h}\\ +\alpha h+\frac{1}{6}\beta h\left(c_{j-1}+4c_{j}+c_{j+1}\right)\\ +\frac{1}{12}\gamma h\left(c_{j-1}^{2}+2c_{j}c_{j-1}+6c_{j}^{2}+c_{j+1}^{2}+2c_{j}c_{j+1}\right)\\ +\frac{1}{20}\delta h\left(c_{j-1}^{3}+2c_{j}c_{j-1}^{2}+3c_{j}^{2}c_{j-1}+8c_{j}^{3}+c_{j+1}^{3}+2c_{j}c_{j+1}^{2}+3c_{j}^{2}c_{j+1}\right)\\ \text{for}\;j=1,2,3,\ldots .n. \end{array}$$

This gives an algebraic system of nonlinear equations. The initial data are

(50) $$\begin{array}{c} c_{0}=0,\\ c_{1}=c_{1}=\frac{T{\dot{q}}_{0}}{n}. \end{array}$$

Observe that algebraic system (39) may be solved *recursively* using the Tartaglia formula for the cubic. Indeed, we may write

(51) $$\begin{array}{c} \frac{1}{20}\delta hz^{3}+\frac{1}{60}h\left(6\delta c_{j}+5\gamma \right)z^{2}+\frac{h^{2}\left(10\beta +10\gamma c_{j}+9\delta c_{j}^{2}\right)+60}{60h}\\ z+\frac{1}{60h}\left(\begin{array}{c} c_{j-1}\left(h^{2}c_{j}\left(9\delta c_{j}+10\gamma \right)+10\left(\beta h^{2}+6\right)\right)+40\beta h^{2}c_{j}+\\ h^{2}c_{j-1}^{2}\left(6\delta c_{j}+5\gamma \right)+6h^{2}c_{j}^{2}\left(4\delta c_{j}+5\gamma \right)+3\delta h^{2}c_{j-1}^{3}-120c_{j}+60\alpha h^{2} \end{array}\right)=0,\\ \text{where}\\ z=c_{j+1}. \end{array}$$

**Example 3 .**

**Figure 4:**
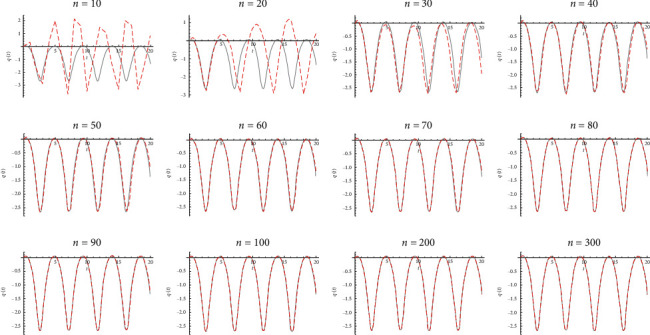
Galerkin hat method for different values of n.

Let *α*=1, *β*=*−*1, *γ*=1, *δ*=1, *q*_0_=0, ${\dot{q}}_{0}=0.3$, and *T*=20 (see Figure 4 for a comparison between the exact solution and the Galerkin method for different values of *n*).

### 3.4. Duffing Equation with Damping and Forcing

Let

(52) $$\begin{array}{c} \ddot{x}+2\varepsilon \dot{x}+\alpha x+\beta x^{3}=F\cos\left(\omega t\right),\\ x\left(0\right)=0,\\ x^{\prime }\left(0\right)={\dot{x}}_{0},\\ 0\leq t\leq T. \end{array}$$

Assume the ansatz

(53) $$\begin{array}{c} x=x\left(t\right)=\sum_{k=1}^{n}c_{k}\varphi_{k}\left(t\right). \end{array}$$

Define *c*_0_=*c*_*n*+1_=0. We have

(54) $$\begin{array}{c} \int_{0}^{T}\left(\ddot{x}+2\varepsilon \dot{x}+\alpha x+\beta x^{3}-F\cos\left(\omega t\right)\right)\varphi_{j}\left(t\right)\text{d}t\\ =\frac{1}{20}\beta hc_{j+1}^{3}+\frac{1}{10}\beta hc_{j}c_{j+1}^{2}+\left(\frac{3}{20}\beta hc_{j}^{2}+\varepsilon +\frac{\alpha h}{6}+\frac{1}{h}\right)\\ c_{j+1}+\frac{1}{60h}\left(\begin{array}{c} 8\left(5c_{j}\left(\alpha h^{2}-3\right)+3\beta h^{2}c_{j}^{3}-\frac{30F}{\omega^{2}}\sin^{2}\left(\frac{h\omega }{2}\right)\cos\left(hj\omega \right)\right)+\\ c_{j-1}\left(9\beta h^{2}c_{j}^{2}+10\left(\alpha h^{2}-6\varepsilon h+6\right)\right)+3\beta h^{2}c_{j-1}^{3}+6\beta h^{2}c_{j}c_{j-1}^{2} \end{array}\right)=0,\\ j=1,2,3,\ldots . \end{array}$$

We may solve this system recursively using the Tartaglia formula for the cubic. The initial data are

(55) $$\begin{array}{c} c_{0}=0,\\ c_{1}=\frac{T}{n}{\dot{x}}_{0}. \end{array}$$

### 3.5. Forced Van der Pol–Duffing Equation

Let

(56) $$\begin{array}{c} \ddot{x}-\varepsilon \left(1-x^{2}\right)\dot{x}+\alpha x+\beta x^{3}=F\cos\left(\omega t\right),\\ x\left(0\right)=0,\\ x^{\prime }\left(0\right)={\dot{x}}_{0},\\ 0\leq t\leq T. \end{array}$$

Assume the ansatz

(57) $$\begin{array}{c} x=x\left(t\right)=\sum_{k=1}^{n}c_{k}\varphi_{k}\left(t\right). \end{array}$$

Define *c*_0_=*c*_*n*+1_=0. We have

(58) $$\begin{array}{c} \int_{0}^{T}\left(\ddot{x}-\varepsilon \left(1-x^{2}\right)\dot{x}+\alpha x+\beta x^{3}-F\cos\left(\omega t\right)\right)\\ \varphi_{j}\left(t\right)\text{d}t=\frac{1}{60}\left(5\varepsilon +3\beta h\right)c_{j+1}^{3}+\frac{1}{60}\;c_{j}\left(5\varepsilon +6\beta h\right)c_{j+1}^{2}+\left(\frac{1}{60}\;c_{j}^{2}\left(5\varepsilon +9\beta h\right)-\frac{\varepsilon }{2}+\frac{\alpha h}{6}+\frac{1}{h}\right)\\ c_{j+1}+\frac{1}{60h}\left(\begin{array}{c} 8\left(5c_{j}\left(\alpha h^{2}-3\right)+3\beta h^{2}c_{j}^{3}-\frac{30F}{\omega^{2}}\sin^{2}\left(\frac{h\omega }{2}\right)\cos\left(hj\omega \right)\right)+\\ c_{j-1}\left(hc_{j}^{2}\left(9\beta h-5\varepsilon \right)+10\left(\alpha h^{2}+3\varepsilon h+6\right)\right)+hc_{j-1}^{3}\left(3\beta h-5\varepsilon \right)+hc_{j}c_{j-1}^{2}\left(6\beta h-5\varepsilon \right) \end{array}\right)=0,\;\text{for}j=1,2,3,\ldots . \end{array}$$

We may solve this system recursively using the Tartaglia formula for the cubic. The initial data are

(59) $$\begin{array}{c} c_{0}=0,\\ c_{1}=\frac{n{\dot{x}}_{0}}{T}. \end{array}$$

### 3.6. Conservative Nonlinear Oscillators

Conservative single-degree-of-freedom nonlinear oscillators are modelled by second-order autonomous ordinary differential equations of the form

(60) $$\begin{array}{c} \ddot{x}+F\left(x\right)=0,\\ x\left(0\right)=x_{0},\\ x^{\prime }\left(0\right)=0. \end{array}$$

Using Chebyshev polynomials or minimization techniques, we may approximate the function *F*(*x*) by means of a cubic polynomial, say *F*(*x*)=*α*+*βx*+*γx*^2^+*δx*^3^, and this allows us to study i.v.p. (44) using the solution to the i.v.p.

(61) $$\begin{array}{c} \ddot{x}+\alpha +\beta x+\gamma x^{2}+\delta x^{3}=0,\\ x\left(0\right)=x_{0},\\ x^{\prime }\left(0\right)=0. \end{array}$$

For example, let us consider a mass attached to two stretched elastic springs [8]. For this problem, the function *F*(*x*) has the form

(62) $$\begin{array}{c} F\left(x\right)=x-\frac{\lambda x}{\sqrt{1+x^{2}}},\;0<\lambda \leq 1. \end{array}$$

Suppose that |*x*| ≤ *A*. We have *F*(*x*) ≈ *px*+*qx*^3^, where

(63) $$\begin{array}{c} p=1+\frac{15\lambda \left(\left(20A^{2}+21\right)\sinh^{-1}\left(A\right)-3A\sqrt{A^{2}+1}\left(2A^{2}+7\right)\right)}{32A^{5}},\\ q=\frac{35\lambda \left(A\sqrt{A^{2}+1}\left(2A^{2}+15\right)-3\left(4A^{2}+5\right)\sinh^{-1}\left(A\right)\right)}{32A^{7}}. \end{array}$$

Then, the problem reduces to that of solving a Duffing equation.

## 4. Analysis and Discussion

We have described the way to solve strongly nonlinear oscillators by means of the Galerkin method. In general, any second-order ordinary differential equation may be solved using the Runge–Kutta numerical method. In general, the Runge-Kutta numerical solution offers a more accurate than the Galerkin solution. On the other hand, the Galerkin method offers the possibility to write the solution as a linear combination of hat functions. In this sense, the Galerkin method is a kind of analytical method. For a given conservative oscillator (45), we may approximate the function *F*(*x*) by a cubic polynomial and then we replace the original problem with problem (45), which has exact analytical solution. However, the exact solution demands the evaluation of either a Jacobian or elliptic Weierstrass function, which has extra costs.

## 5. Conclusions

The Galerkin method offers a way to obtain semianalytical solution to a given nonlinear oscillator of special form (1). The exact solution to it is not known in general. There are other ways to solve it. Perturbative methods like the Lindstedt–Poincaré method or the Krylov–Bogoliubov–Mitropolsky method are also possible for this end. The advantage of the proposed method consists of the possibility to solve the Galerkin equations iteratively using an algebraic equation. The Galerkin hat method is usually used to solve second-order linear ODEs. In this work, we extended it to a class of nonlinear oscillators. Other methods for solving nonlinear differential equations may be found in [8–10].

## Data Availability

No data were used to support this study.

## Appendix

### Formulas for Computing

(A.1)
$$\begin{array}{c} {\text{ true}}_{0}^{T}t^{\rho }x\left(t\right)\varphi_{j}\left(t\right)\text{d}t=\frac{Tn^{-2\rho -1}}{\left(\rho +1\right)\left(\rho +2\right)\left(\rho +3\right)},\\ \left(\begin{array}{c} c_{j-1}n^{\rho }\left({\left(j-1\right)}^{2}\left(2j+\rho +1\right){\left(\left(j-1\right)T\right)}^{\rho }-j^{2}\left(2j-\rho -3\right){\left(jT\right)}^{\rho }\right)+\\ c_{j+1}n^{\rho }\left(j^{2}\left(2j+\rho +3\right){\left(jT\right)}^{\rho }+{\left(j+1\right)}^{2}\left(-2j+\rho +1\right){\left(\left(j+1\right)T\right)}^{\rho }\right)+\\ 2c_{j}\left(\begin{array}{c} j\left(-2j\left(\rho +3\right)n^{\rho }+\left(-\left(j-3\right)j-3\right){\left(\frac{\left(j-1\right)n}{j}\right)}^{\rho }+\left(j\left(j+3\right)+3\right){\left(\frac{n}{j}+n\right)}^{\rho }\right)+\\ {\left(\frac{\left(j-1\right)n}{j}\right)}^{\rho }+{\left(\frac{n}{j}+n\right)}^{\rho } \end{array}{\left(jT\right)}^{\rho }\right) \end{array}\right),\\ \int_{0}^{T}t^{\rho }x{\left(t\right)}^{2}\varphi_{j}\left(t\right)dt=\frac{T}{\left(\rho +1\right)\left(\rho +2\right)\left(\rho +3\right)\left(\rho +4\right)n^{2\rho +1}}\\ \left(\left(\left(\begin{array}{c} 2c_{j-1}c_{j}n^{\rho }\begin{array}{c} \left(j^{2}\left(6j^{2}-4j\left(\rho +4\right)+\left(\rho +3\right)\left(\rho +4\right)\right){\left(jT\right)}^{\rho }-\right)\\ 2{\left(j-1\right)}^{3}\left(3j+\rho +1\right){\left(\left(j-1\right)T\right)}^{\rho } \end{array}+\\ 2c_{j}c_{j+1}n^{\rho }\left(\begin{array}{c} j^{2}\left(6j^{2}+4j\left(\rho +4\right)+\left(\rho +3\right)\left(\rho +4\right)\right){\left(jT\right)}^{\rho }+\\ 2{\left(j+1\right)}^{3}\left(-3j+\rho +1\right){\left(\left(j+1\right)T\right)}^{\rho } \end{array}\right)+\\ 6c_{j}^{2}\left(\begin{array}{c} j\left(\begin{array}{c} j\left(\begin{array}{c} -\left(2j^{2}+\left(\rho +3\right)\left(\rho +4\right)\right)n^{\rho }+\left(\left(j-4\right)j+6\right){\left(\frac{\left(j-1\right)n}{j}\right)}^{\rho }+\\ \left(j\left(j+4\right)+6\right){\left(\frac{n}{j}+n\right)}^{\rho } \end{array}\right)+\\ 4\left({\left(\left(\frac{1}{j}+1\right)n\right)}^{\rho }-{\left(\frac{\left(j-1\right)n}{j}\right)}^{\rho }\right) \end{array}\right)+\\ {\left(\left(\frac{1}{j}+1\right)n\right)}^{\rho }+{\left(\frac{\left(j-1\right)n}{j}\right)}^{\rho } \end{array}\right)\\ {\left(jT\right)}^{\rho }+c_{j-1}^{2}n^{\rho }\left({\left(j-1\right)}^{2}\left(6j^{2}+4j\left(\rho +1\right)+\left(\rho +1\right)\left(\rho +2\right)\right){\left(\left(j-1\right)T\right)}^{\rho }-2j^{3}\left(3j-\rho -4\right){\left(jT\right)}^{\rho }\right)+\\ c_{j+1}^{2}n^{\rho }\left({\left(j+1\right)}^{2}\left(6j^{2}-4j\left(\rho +1\right)+\left(\rho +1\right)\left(\rho +2\right)\right){\left(\left(j+1\right)T\right)}^{\rho }-2j^{3}\left(3j+\rho +4\right){\left(jT\right)}^{\rho }\right) \end{array}\right)\right)\right),\\ \int_{0}^{T}t^{\rho }x{\left(t\right)}^{3}\varphi_{j}\left(t\right)\text{d}t=\frac{T}{\left(\rho +1\right)\left(\rho +2\right)\left(\rho +3\right)\left(\rho +4\right)\left(\rho +5\right)n^{2\rho +1}}\\ \left(\begin{array}{c} 8c_{j}^{3}\left(\begin{array}{c} j\left(\begin{array}{c} -j\left(\rho +5\right)\left(6j^{2}+\left(\rho +3\right)\left(\rho +4\right)\right)n^{\rho }+\\ 3\left(j\left(j\left(j\left(j+5\right)+10\right)+10\right)+5\right){\left(\left(\frac{1}{j}+1\right)n\right)}^{\rho }-\\ 3\left(j\left(j\left(\left(j-5\right)j+10\right)-10\right)+5\right){\left(\frac{\left(j-1\right)n}{j}\right)}^{\rho } \end{array}\right)+\\ 3\left({\left(\left(\frac{1}{j}+1\right)n\right)}^{\rho }+{\left(\frac{\left(j-1\right)n}{j}\right)}^{\rho }\right) \end{array}\right)\\ {\left(jT\right)}^{\rho }+3c_{j-1}c_{j}^{2}n^{\rho }\left(\begin{array}{c} 6{\left(j-1\right)}^{4}\left(4j+\rho +1\right){\left(\left(j-1\right)T\right)}^{\rho }-\\ j^{2}\left(\begin{array}{c} 24j^{3}-18j^{2}\left(\rho +5\right)+\\ 6j\left(\rho +4\right)\left(\rho +5\right)-\left(\rho +3\right)\left(\rho +4\right)\left(\rho +5\right) \end{array}\right){\left(jT\right)}^{\rho } \end{array}\right)+\\ 6c_{j}c_{j+1}^{2}n^{\rho }\left(\begin{array}{c} {\left(j+1\right)}^{3}\left(12j^{2}-6j\left(\rho +1\right)+\left(\rho +1\right)\left(\rho +2\right)\right){\left(\left(j+1\right)T\right)}^{\rho }-\\ j^{3}\left(\begin{array}{c} 12j^{2}+6j\left(\rho +5\right)+\\ \left(\rho +4\right)\left(\rho +5\right) \end{array}\right){\left(jT\right)}^{\rho } \end{array}\right)+\\ 6c_{j-1}^{2}c_{j}n^{\rho }\left(\begin{array}{c} j^{3}\left(12j^{2}-6j\left(\rho +5\right)+\left(\rho +4\right)\left(\rho +5\right)\right){\left(jT\right)}^{\rho }-\\ {\left(j-1\right)}^{3}\left(\begin{array}{c} 12j^{2}+6j\left(\rho +1\right)+\\ \left(\rho +1\right)\left(\rho +2\right) \end{array}\right){\left(\left(j-1\right)T\right)}^{\rho } \end{array}\right)+\\ 3c_{j}^{2}c_{j+1}n^{\rho }\left(\begin{array}{c} j^{2}\left(24j^{3}+18j^{2}\left(\rho +5\right)+6j\left(\rho +4\right)\left(\rho +5\right)+\left(\rho +3\right)\left(\rho +4\right)\left(\rho +5\right)\right){\left(jT\right)}^{\rho }+\\ 6{\left(j+1\right)}^{4}\left(-4j+\rho +1\right){\left(\left(j+1\right)T\right)}^{\rho } \end{array}\right)+\\ c_{j-1}^{3}n^{\rho }\left({\left(j-1\right)}^{2}\left(\begin{array}{c} 24j^{3}+18j^{2}\left(\rho +1\right)+6j\left(\rho +1\right)\left(\rho +2\right)+\\ \left(\rho +1\right)\left(\rho +2\right)\left(\rho +3\right) \end{array}\right){\left(\left(j-1\right)T\right)}^{\rho }-6j^{4}\left(4j-\rho -5\right){\left(jT\right)}^{\rho }\right)+\\ c_{j+1}^{3}n^{\rho }\left(6j^{4}\left(4j+\rho +5\right){\left(jT\right)}^{\rho }+{\left(j+1\right)}^{2}\left(\begin{array}{c} -24j^{3}+18j^{2}\left(\rho \right.+1\left.-\right)\\ 6j\left(\rho +1\right)\left(\rho +2\right)+\left(\rho +1\right)\left(\rho +2\right)\left(\rho +3\right) \end{array}\right){\left(\left(j+1\right)T\right)}^{\rho }\right) \end{array}\right),\\ \int_{0}^{T}\sin\left(\omega t\right)x\left(t\right)\varphi_{j}\left(t\right)\text{d}t\\ =\frac{-4c_{j}\left(\sin\left(h\omega \right)-h\omega \right)\sin\left(hj\omega \right)+c_{j-1}\left(-h\omega \sin\left(h\left(j-1\right)\omega \right)+\sin\left(hj\omega \right)\right)+\left.2\cos\left(h\left(j-1\right)\omega \right)-2\cos\left(hj\omega \right)\right)+c_{j+1}\left(-h\omega \left(\sin\left(hj\omega \right)+\sin\left(h\left(j\right.+1\left.\omega \right)\right)\right)+2\cos\left(hj\omega \right)-2\cos\left(h\left(j+1\right)\omega \right)\right)}{h^{2}\omega^{3}},\\ \int_{0}^{T}\cos\left(\omega t\right)x\left(t\right)\varphi_{j}\left(t\right)\text{d}t=-\frac{\begin{array}{c} c_{j-1}\left(2\left(\sin\left(h\left(j-1\right)\omega \right)-\sin\left(hj\omega \right)+h\omega \cos\left(h\left(j-1\right)\omega \right)+\cos\left(hj\omega \right)\right)\right)+\\ 4c_{j}\left(\sin\left(h\omega \right)-h\omega \right)\cos\left(hj\omega \right)+c_{j+1}\left(2\left(\sin\left(hj\omega \right)-\sin\left(h\left(j+1\right)\omega \right)\right)+h\omega \left(\cos\left(hj\omega \right)+\cos\left(h\left(j+1\right)\omega \right)\right)\right) \end{array}}{h^{2}\omega^{3}}\\ \int_{0}^{T}\exp\left(x\left(t\right)\right)\varphi_{j}\left(t\right)\text{d}t=\frac{T^{2}\left(\left(e^{c_{j}}-e^{c_{j+1}}\right)c_{j-1}+\left(e^{c_{j-1}}-2e^{c_{j}}+e^{c_{j+1}}\right)c_{j}-\left(e^{c_{j-1}}-e^{c_{j}}\right)c_{j+1}\right)}{n^{2}\left(c_{j-1}-c_{j}\right)\left(c_{j}-c_{j+1}\right)},\\ \int_{0}^{T}x\left(t\right)x^{\prime }\left(t\right)\varphi_{j}\left(t\right)\text{d}t=-\frac{1}{6}\left(c_{j-1}-c_{j+1}\right)\left(c_{j-1}+c_{j}+c_{j+1}\right),\\ \int_{0}^{T}x{\left(t\right)}^{2}x^{\prime }\left(t\right)\varphi_{j}\left(t\right)\text{d}t=\frac{1}{12}\left(c_{j+1}^{3}+c_{j}c_{j+1}^{2}+c_{j}^{2}c_{j+1}-c_{j-1}\left(c_{j-1}^{2}+c_{j}c_{j-1}+c_{j}^{2}\right)\right),\\ \int_{0}^{T}x{\left(t\right)}^{3}x^{\prime }\left(t\right)\varphi_{j}\left(t\right)\text{d}t=\frac{1}{20}\left(c_{j+1}^{4}+c_{j}c_{j+1}^{3}+c_{j}^{2}c_{j+1}^{2}+c_{j}^{3}c_{j+1}-c_{j-1}\left(c_{j-1}+c_{j}\right)\left(c_{j-1}^{2}+c_{j}^{2}\right)\right),\\ \int_{0}^{T}x\left(t\right)x^{\prime }{\left(t\right)}^{2}\varphi_{j}\left(t\right)\text{d}t=\frac{n\left(c_{j-1}^{3}-3c_{j}^{2}c_{j-1}+4c_{j}^{3}+c_{j+1}^{3}-3c_{j}^{2}c_{j+1}\right)}{6T},\\ \int_{0}^{T}x{\left(t\right)}^{2}x^{\prime }{\left(t\right)}^{2}\varphi_{j}\left(t\right)\text{d}t=\frac{n\left(c_{j-1}^{4}-4c_{j}^{3}c_{j-1}+6c_{j}^{4}+c_{j+1}^{4}-4c_{j}^{3}c_{j+1}\right)}{12T},\\ \int_{0}^{T}x{\left(t\right)}^{3}x^{\prime }{\left(t\right)}^{2}\varphi_{j}\left(t\right)\text{d}t=\frac{1}{20}\left(c_{j+1}^{4}+c_{j}c_{j+1}^{3}+c_{j}^{2}c_{j+1}^{2}+c_{j}^{3}c_{j+1}-c_{j-1}\left(c_{j-1}+c_{j}\right)\left(c_{j-1}^{2}+c_{j}^{2}\right)\right),\\ \int_{0}^{T}x{\left(t\right)}^{3}x^{\prime }\left(t\right)\varphi_{j}\left(t\right)\text{d}t=\frac{n\left(c_{j-1}^{5}-5c_{j}^{4}c_{j-1}+8c_{j}^{5}+c_{j+1}^{5}-5c_{j}^{4}c_{j+1}\right)}{20T},\\ \int_{0}^{T}x{\left(t\right)}^{4}x^{\prime }\left(t\right)\varphi_{j}\left(t\right)\text{d}t=\frac{1}{30}\left(c_{j+1}^{5}+c_{j}c_{j+1}^{4}+c_{j}^{2}c_{j+1}^{3}+c_{j}^{3}c_{j+1}^{2}+c_{j}^{4}c_{j+1}-c_{j-1}\left(c_{j-1}^{4}+c_{j}c_{j-1}^{3}+c_{j}^{2}c_{j-1}^{2}+c_{j}^{3}c_{j-1}+c_{j}^{4}\right)\right)\\ \int_{0}^{T}x{\left(t\right)}^{5}x^{\prime }\left(t\right)\varphi_{j}\left(t\right)\text{d}t=\frac{1}{42}\left(\begin{array}{c} c_{j+1}^{6}+c_{j}c_{j+1}^{5}+c_{j}^{2}c_{j+1}^{4}+c_{j}^{3}c_{j+1}^{3}+c_{j}^{4}c_{j+1}^{2}+c_{j}^{5}c_{j+1}-\\ c_{j-1}\left(c_{j-1}+c_{j}\right)\left(c_{j-1}^{2}-c_{j}c_{j-1}+c_{j}^{2}\right)\left(c_{j-1}^{2}+c_{j}c_{j-1}+c_{j}^{2}\right) \end{array}\right),\\ \int_{0}^{T}\varphi_{j}\left(t\right)\cos\left(t\omega \right)\text{d}t=\frac{2n\left(1-\cos\left(T\omega /n\right)\right)\cos\left(jT\omega /n\right)}{T\omega^{2}},\\ \int_{0}^{T}x{\left(t\right)}^{5}x^{\prime }\left(t\right)\varphi_{j}\left(t\right)\text{d}t=\frac{1}{42}\left(\begin{array}{c} c_{j+1}^{6}+c_{j}c_{j+1}^{5}+c_{j}^{2}c_{j+1}^{4}+c_{j}^{3}c_{j+1}^{3}+c_{j}^{4}c_{j+1}^{2}+c_{j}^{5}c_{j+1}-\\ c_{j-1}\left(c_{j-1}+c_{j}\right)\left(c_{j-1}^{2}-c_{j}c_{j-1}+c_{j}^{2}\right)\left(c_{j-1}^{2}+c_{j}c_{j-1}+c_{j}^{2}\right) \end{array}\right),\\ \int_{0}^{T}\varphi_{j}\left(t\right)\cos\left(t\omega \right)\text{d}t=\frac{2n\left(1-\cos\left(T\omega /n\right)\right)\cos\left(jT\omega /n\right)}{T\omega^{2}}. \end{array}$$
